# The Dietary Fiber Pectin: Health Benefits and Potential for the Treatment of Allergies by Modulation of Gut Microbiota

**DOI:** 10.1007/s11882-021-01020-z

**Published:** 2021-09-10

**Authors:** Frank Blanco-Pérez, Hanna Steigerwald, Stefan Schülke, Stefan Vieths, Masako Toda, Stephan Scheurer

**Affiliations:** 1 Molecular Allergology, Federal Institute for Vaccines and Biomedicines, Paul-Ehrlich-Institut, Langen, Germany; 2 Laboratory of Food and Biomolecular Science, Graduate School of Agricultural Science, Tohoku University, Sendai, Japan

**Keywords:** Pectin, Dietary fiber, Prebiotics, Microbiota, SCFA, Allergy

## Abstract

**Purpose of Review:**

The incidence of allergies is increasing and has been associated with several environmental factors including westernized diets. Changes in environment and nutrition can result in dysbiosis of the skin, gut, and lung microbiota altering the production of microbial metabolites, which may in turn generate epigenetic modifications. The present review addresses studies on pectin-mediated effects on allergies, including the immune modulating mechanisms by bacterial metabolites.

**Recent Findings:**

Recently, microbiota have gained attention as target for allergy intervention, especially with prebiotics, that are able to stimulate the growth and activity of certain microorganisms. Dietary fibers, which cannot be digested in the gastrointestinal tract, can alter the gut microbiota and lead to increased local and systemic concentrations of gut microbiota*-*derived short chain fatty acids (SCFAs). These can promote the generation of peripheral regulatory T cells (T_reg_) by epigenetic modulation and suppress the inflammatory function of dendritic cells (DCs) by transcriptional modulation.

The dietary fiber pectin (a plant-derived polysaccharide commonly used as gelling agent and dietary supplement) can alter the ratio of *Firmicutes* to *Bacteroidetes* in gut and lung microbiota, increasing the concentrations of SCFAs in feces and sera, and reducing the development of airway inflammation by suppressing DC function.

**Summary:**

Pectin has shown immunomodulatory effects on allergies, although the underlying mechanisms still need to be elucidated. It has been suggested that the different types of pectin may exert direct and/or indirect immunomodulatory effects through different mechanisms. However, little is known about the relation of certain pectin structures to allergies.

## Introduction

The manifestation of allergies frequently is associated with a dysbiosis of the gut microbiome, which can be affected by environmental factors, cesarean section, antiseptic agents, lack of breastfeeding, certain drugs, and a low-fiber/high-fat diet [1, 2]. In contrast, homeostasis of gut microbiota can be achieved by the intake of prebiotics [3, 4]. The International Scientific Association for Probiotics and Prebiotics (ISAPP) defined prebiotics as “a substrate that is selectively fermented by a host microorganism that allows specific changes, both in the composition and/or activity in the gastrointestinal microflora that confers benefits upon host well-being and health” [5–7]. To be classified as prebiotics, the compounds must meet the following criteria: (I) non-digestible and resistant to breakdown by stomach acid and enzymes in the human gastrointestinal tract, (II) selectively fermented by intestinal microorganisms of the host, and (III) selectively targeting and stimulating the growth and activity of beneficial bacteria in the gut [8, 9].

Prebiotics beneficially influence the health of the host by either (I) indirectly increasing the secretion of bacteria-derived metabolites into the intestinal tract, in turn influencing many molecular and cellular processes, or by (II) directly affecting the immune response of certain cells, e.g., epithelial and immune cells [10]. Therefore, prebiotics are considered as an immune active agent conferring a health benefit [11, 12].

**Fig. 1:**
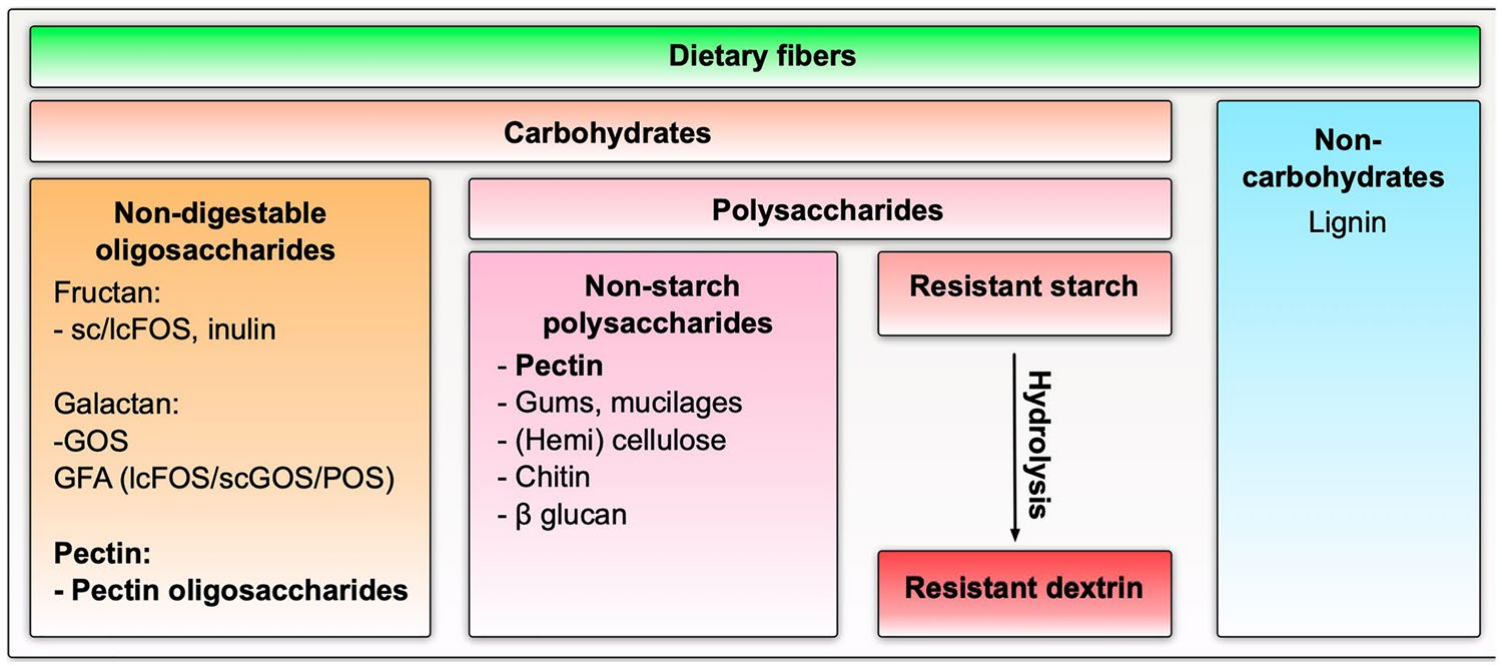
Dietary fibers (overview). Dietary fibers are polymers mainly derived from edible parts of plants, certain types of animals or analogous carbohydrates that are neither digested nor absorbed in the human intestine. They can be divided into carbohydrates and non-carbohydrates (e.g., lignin). Carbohydrate fibers can be further subdivided in non-digestible oligosaccharides or polysaccharides such as non-starch-, resistant starch-, or dextran polysaccharides

Dietary fibers are polymers with three or more monomeric units (MU) which are mainly derived from edible parts of plants, certain types of animals (e.g., crustacean), or analogous carbohydrates that are neither digested nor absorbed in the human intestine [13]. Therefore, they pass through the upper part of the gastrointestinal tract into the large intestine where they are fermented by advantageous bacteria stimulating their growth and activity, which confer them prebiotic activity [12, 14, 15]. Dietary fibers consist of carbohydrates as non-digestible oligosaccharides (e.g., short-chain and long-chain fructooligosaccharides (sc/lc FOS) and galactooligosaccharides (GOS), inulin) [16]; non-starch polysaccharides such as pectin, chitins, beta-glucan; and other plant components such as cellulose [17–19], resistant starch [20], or resistant dextrin [21]. They also consist of non-carbohydrates, like lignin, that can also act as a dietary fiber (Fig. 1).

Dietary fibers confer health benefits comprising decreased risks of coronary heart disease, colon cancer, and type 2 diabetes. Clinically, fiber deficiency increases the risk of colon, liver, and breast cancer, and increases mortality and death from both cancerous and non-cancerous diseases [22].

Supplementation of prebiotics as food ingredients has been proposed to prevent several inflammatory diseases [23–27] as well as allergies [10, 28, 29, 30, 31, 32]. Cohort studies have indicated that one of the factors preceding the development of food allergies is gut dysbiosis [2, 33, 34]. Therefore, gut microbiota have gained attention as a target of intervention against allergies, especially with prebiotics.

So far, most studies exploring the effect of dietary fiber on the allergic immune response used non-digestible oligosaccharides [10, 35, 36]. Human milk–derived non-digestible oligosaccharides comprise short-chain galacto- (scGOS), long-chain fructo- (lsFOS), and acidic pectin–derived oligosaccharides (pAOS), which are together referred to as galacto-, fructo-, and acidic oligosaccharides (GFAs). Dietary fibers undergo microbial fermentation by commensal gut bacteria producing short-chain fatty acids (SCFA) with immune modulating properties [37, 38]. Long-term deficiency of dietary fiber intake increases the susceptibility to airway allergic disease (AAD), whereas proper fiber supplementation effectively promotes balanced Th1/Th2 immunity, significantly attenuates allergic inflammatory responses, and optimizes the structure of intestinal microbiota, which suggests its potential for novel preventive and therapeutic intervention strategies [39]. Taking this into consideration, this review gives an in-depth overview of the reported effects of dietary fiber pectin on the immunomodulation of allergic diseases.

## Pectin: Characteristics and Immune Modulating Effects

### Pectin Structure

**Fig. 2:**
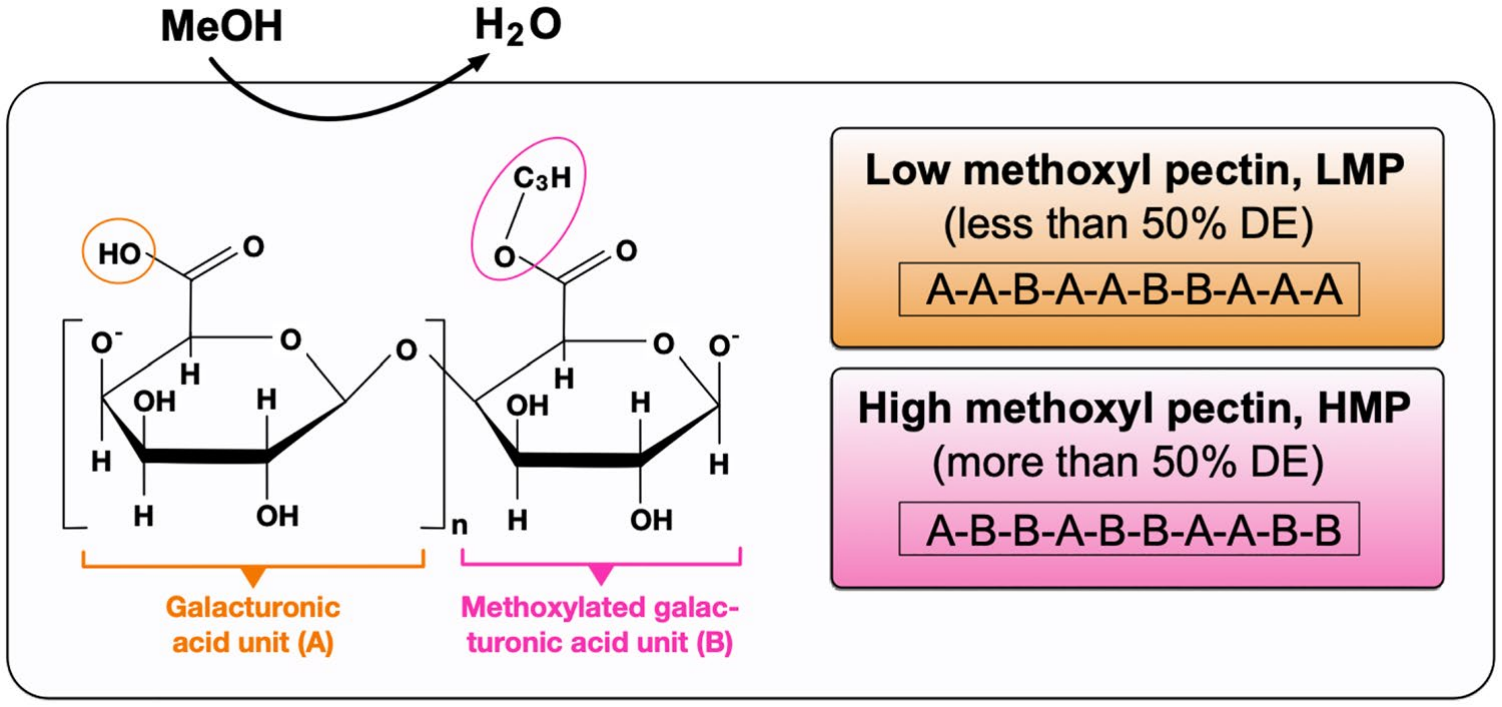
Pectin structure and health benefits. Pectin consists of a linear backbone of at least 65% galacturonic acid (GalA) that can be methyl-esterified at the carboxyl groups present at C-6. The ratio of esterified to non-esterified GalA groups is termed the degree of esterification (DE). Pectin are classified as high methoxy pectin (HMP) with DE > 50% or low methoxy pectin (LMP) with DE < 50%

Pectin is a dietary fiber accumulating in the primary cell walls and intercellular tissues of terrestrial plants, where it plays an important role as hydrating agent and cementing material [40, 41]. Pectin is a heterogeneous and complex acidic hetero-polysaccharide with a molecular mass of typically 50,000–150,000 g/mol, depending on extraction method and source material [42]. It contains a linear backbone of at least 65% galacturonic acid (GalA), which can either be free or methyl-esterified at the carboxyl groups present at C-6 (Fig. 2) [43, 44]. The pectin macromolecule contains fragments of linear and branched regions of polysaccharides such as homogalacturonan, rhamnogalacturonan, xylogalacturonan, and apiogalacturonan [45, 46]. In native, non-processed pectin, approximately 80% of carboxyl groups of GalA are esterified with methanol and present as methyl esters. Thus, the ratio of esterified GalA groups to total GalA groups is termed the degree of esterification (DE). Pectins are classified as high methoxy pectin (HMP) with DE > 50% or low methoxy pectin (LMP) with DE < 50% (Fig. 2) [44]. The majority of natural pectins is HMP (~ 80% DE), whereas LMP is more common in processed foods [47, 48]. The degree of esterification determines the properties of pectin in food technology as HMP can form a gel under acidic conditions (pH ~ 3) in the presence of high sugar concentrations, while LMP form gels by interaction with divalent cations, particularly Ca^2+^ [47, 48]. Both HMP and LMP appear to possess immunomodulatory effects in mice. LMP likely is more efficiently fermented by the microbiota in the ileum, whereas HMP is mainly fermented in the proximal colon [49]. Moreover, it is suggested that structural features determine the effect of pectin on the immune system. Evidence has been provided that the backbones of pectin macromolecules have immunosuppressive activity [50].

Depolymerization of purified pectin or the raw materials by partial enzymatic hydrolysis leads to production of Pectin-derived oligosaccharides (POS) which were indicated as new prebiotic candidates [51]. The main suggested properties of POS stimulation are (I) growth of beneficial bacteria in the colon, (II) apoptosis of colon cancer cells, and (III) protection again various pathogens [51]. The different POS can include galacturonic acid (GalA), rhamnose (Rha), arabinose (Ara), and galactose (Gal) [52]. GFAs, consisting of pAOS, have shown to lower immune responses in cow-milk-allergic (CMA) mice, to enhance regulatory T cell (T_reg_) frequencies, and to induce mucosal IL-10 and TGF-β transcription while suppressing the allergic effector response [53]. Both animal studies and human clinical trials showed that dietary intervention with these dietary oligosaccharides early in life could lead to the prevention of atopic dermatitis, food allergy, and allergic asthma [10, 37, 54, 55]. In line with this, supplementation with two mixtures of scGOS/lcFOS or scGOS/lcFOS/pAOS decreased the OVA-induced airway inflammation and hyperresponsiveness in mice [56]. Moreover, OVA-specific IgE titers were decreased by more than 25%, although this effect was not significant [56]. The effects of the oligosaccharide mixture containing pAOS appeared to be more pronounced than the effects of the scGOS/lcFOS mixture without pAOS [56].

### Pectin-Mediated Health-Promoting Effects

Pectin and dietary fibers in general are considered to provide diverse health benefits including slow gastric emptying [57], improvement of physical bowel function [58], reduced glucose and cholesterol absorption [59], and increase of fecal mass [60, 61]. Pectin is recognized as a prebiotic that is not degraded by either human saliva or gastric acid and is resistant to pepsin, trypsin, and rennet [62, 63]. Several studies demonstrated that pectins from different sources such as apple [64] or citrus [65] can serve as valuable carbon sources for gut bacteria [66•]. The ability to degrade pectins seems to be a common trait among Gram-negative *Bacteroides* species in the human colon [67], whereas only few Gram-positive bacterial species like *Firmicutes* seem to ferment either pectin or its breakdown products [68]. Pectin is fermented by beneficial microbiota mainly in the large intestine (colon), generating the SCFAs acetate, propionate, and butyrate, all of which have beneficial health effects [69, 70]. Evidence suggests that SCFAs can affect the epigenome through metabolic regulatory receptors, potentially reducing obesity, diabetes, atherosclerosis, mucosal inflammation, carcinogenesis, and allergy [22, 71, 72, 73, 74].

The average daily intake of pectin from fruit and vegetables has been estimated to be around 5 g, considering a fruit and vegetable consumption of 500 g per day [75]. Several studies reported that high-fiber diets improve diabetic control via decrease of both glucose and cholesterol absorption as well as lowered serum triglyceride levels [76, 77]. It was found that several dietary fibers lead to delayed absorption of glucose and fatty acids from the upper small intestine, decreasing available substrates for triglyceride synthesis [78]. In humans, pectin consumption (15 g/day over a period of 4 weeks) has been shown to slightly reduce blood LDL cholesterol levels by 3–7% [79, 80]. This effect likely depends on the source of pectin, since apple and citrus pectin were found to be more effective than orange pulp fiber pectin [79]. However, the mechanism appears to be related to an increase of viscosity in the intestinal tract, leading to a reduced absorption of cholesterol from either bile or food [75, 81]*.* Studies propose that the SCFA propionate leads to activation of the adenosine monophosphate-activated protein kinase (AMPK), which is one of the regulators for glucose metabolism in the liver [65, 82-84]. Activated AMPK inhibits acetyl-CoA carboxylase (ACC) leading to a decrease of lipogenesis [65]. Other studies in Apo E–deficient mice comparing high-cholesterol diet (HCD) with or without pectin supplementation showed improved lipid profiles and reduced atherosclerotic plaques in the HCD/pectin group [81]. The study suggested that microbiota-dependent butyrate production inhibits intestinal cholesterol absorption, leading to decreased levels of atherosclerosis [85].

Furthermore, pectin has favorable effects on maintaining the intestinal barrier, which consists of a thick mucus layer protecting the intestinal epithelial tissue. Recent studies found that intervention with pectin in mice led to increased amount of colonic gel-forming mucin 2 (MUC2), the expression of which is related to both the thickness of mucus layer and gut health [86].

Supplementation with LMP reduced type 1 diabetes (T1D) incidence in non-obese diabetic (NOD) mice by positively impacting cecal microbiota, enhancing the production of immune modulating bacterial SCFAs, as well as improving intestinal integrity in the cecum [87]. The maintenance of gut homeostasis by LMP further results in modulated gut-pancreatic autoimmune responses and in protection against T1D development [87]. Other studies revealed that pectin supplementation improved insulin and glucose profiles and reverse calorie restriction (CR)–induced insulin resistance in the rat CR model [88]. By suppressing pro-inflammatory cytokine production, LMP was shown to have anti-inflammatory effects [89]. A fiber-rich diet was also shown to improve glycemic control in patients with type 2 diabetes mellitus [90].

It is suggested that pectin binds metals in the digestive tract, preventing their absorption [91]. Consequently, orally administered pectin is known to (I) remove heavy metals, (II) decrease lead absorption, and (III) reduce strontium bone and blood levels [92, 93].

Furthermore, pectin shows beneficial anti-cancer effects. Studies revealed that pH-modified citrus pectins (MCP), rich in galactoside residues, significantly diminish the number of lung metastases in C57BL/6 mice [94, 95]. This suggests that the galactoside residues impair cell–cell interactions by competing with the endogenous ligands of galactoside-binding proteins such as galectin-3 [94, 95]. Further studies found that modified apple pectin induces apoptosis in colorectal cancer cells by a dose-dependent increase of caspase 3, -8, and -9 expression [96].

A further health claim associated with pectin affects Alzheimer’s disease. A recent study suggests an impact of pectin polysaccharide on Aβ_42_, an important molecule for pathology of Alzheimer’s disease, by inhibition of its aggregation [97].

Moreover, it has been suggested that pectin has beneficial effects on the manifestations of IgE-mediated food and respiratory allergy [98••].

### Effect of Pectin on the Immune Response and Allergic Sensitization

**Fig. 3:**
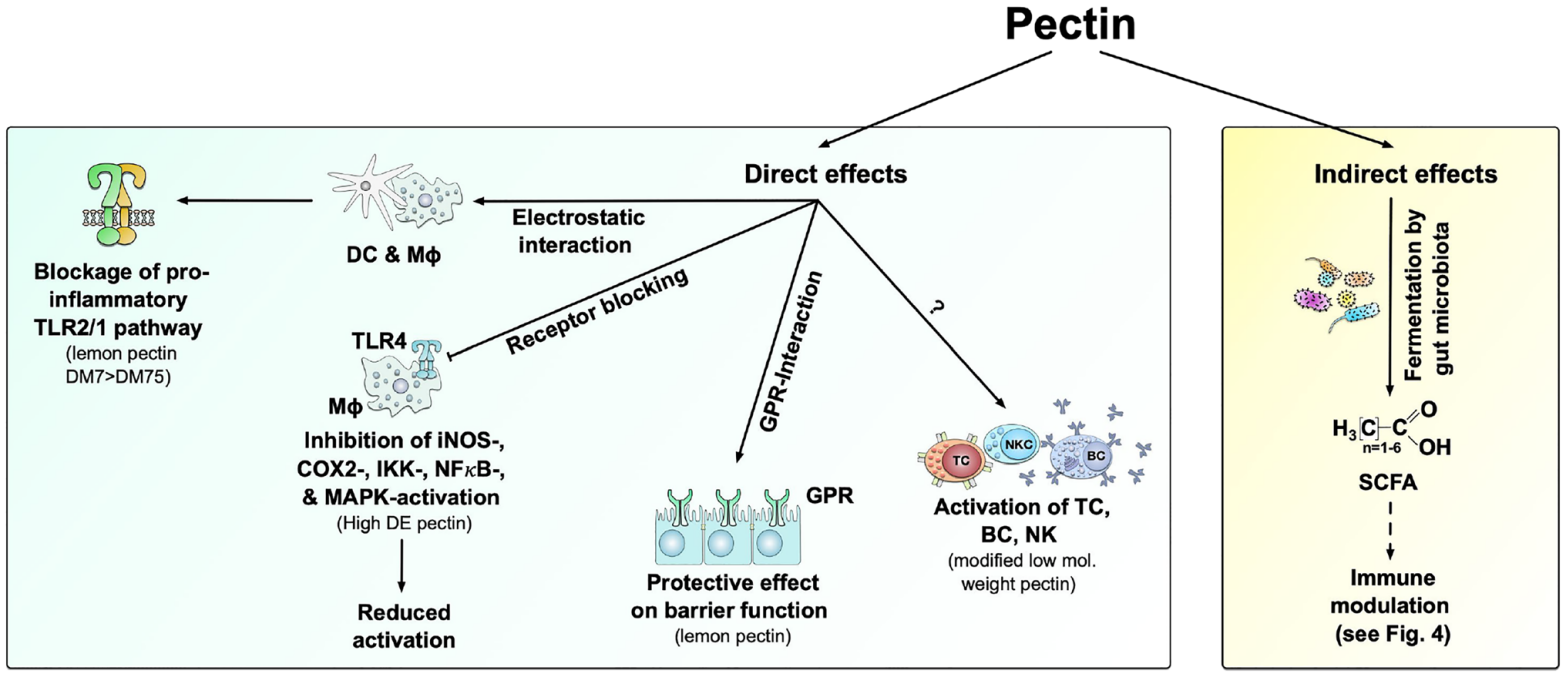
Immune modulation by pectin. Immune modulation by dietary fibers can either be indirectly mediated by their fermentation into short chain fatty acids (SCFAs) or directly caused by the pectin, e.g., via the blockage of the pro-inflammatory TLR2/1 pathway. Several positive health effects are associated with consumption of pectin such as maintaining the intestinal barrier, immune modulation like the activation of immune cells (T, B, NK cells), and the inhibition of inflammatory responses

Pectin displays diverse immunomodulatory properties, comprising both direct effects on immune cells and indirect effects mediated by bacterial metabolites upon fermentation of pectin in the gut (Fig. 3) [99, 100•].

Dietary fibers can directly interact with intestinal cells and mucosal immune cells [101], affecting immune cell responses by interaction with pattern recognition receptors (PRRs). Thereby distinct binding capacities of different pectins may cause the reported differences in immunomodulatory efficiency [102, 103]. The best characterized PRRs in the intestine are Toll-like receptors (TLRs), which were shown to also recognize dietary fibers [104, 105]. It was elucidated in human DCs and the mouse macrophage cell line RAW264.7 in vitro that pectin binds the ectodomain of Toll-like receptor 2 (TLR2) by electrostatic interactions and specifically inhibits the pro-inflammatory TLR2-TLR1 pathway while the tolerogenic TLR2-TLR6 pathway remains unaffected (Fig. 3) [105]. This effect was predominantly achieved with pectin having a low DE [105].

The immunomodulatory effects of pectin mainly depend on the content of galacturonic acid residues and the DE. Pectin with DE up to 80–90% inhibited iNOS and COX2 expression in murine peritoneal macrophages and inhibited MAPK phosphorylation, IKK kinase activity, and NF-κB activation more efficiently than pectin with lower DE (Fig. 3) [106]. In this context, highly esterified pectin was able to bind LPS, modifying its binding to TLR4 [50, 106]. Additionally, MCP with a decreased molecular weight and DE was shown to activate cytotoxic T cells, B cells, as well as NK cells in a dose-dependent manner [107]. Comparing the immunomodulatory effects of native and modified pectin on the example of citrus pectin showed, both types of pectin led to upregulated levels of IFN-γ in the spleen [108]. MCP, but also native citrus pectin, led to increased levels of TNF-a-, IFN-γ-, and IL-17 secretion, likely regulated by IL-4 [108]. Unbranched galacturonan regions were shown to increase the anti-inflammatory properties of pectin [109].

The role of pectin in the manifestation of type 1 allergies is controversially discussed. Different pectins are described to either promote or prevent allergies [98••, 110–114]. The matrix effect of pectin-rich fruits has been suggested to reduce the digestibility of food allergens and thereby to facilitate the process of allergic sensitization in atopic individuals [115]. In line with this, the addition of apple pectin to purified kiwi allergen was able to protect the allergen from pepsin digestion in vitro [115]. An independent study showed that pectin reduces the accessibility of cleavage sites and/or epitope sequences of β-lactoglobulin through a non-specific interaction [116]. Other studies also reported anaphylaxis induced by pectin (Table 1) [110, 111, 113]. Pectin-mediated allergy was reported after drinking a pectin-containing smoothie [110]. In line with this, a positive skin prick test to both pectin and cashew was reported, and cross-reactivity between pectin and cashew was considered [110, 114]. Reports from the 1990s found that continued airborne exposure to pectin in the workplace was associated with the development of occupational asthma [117–119]. Jaakkola et al. reported 3 patients that developed occupational asthma after frequent inhalation of pectin [119]. Two patients showed immediate reduction in lung functions after an inhalation challenge with pectin and positive SPT, while the third showed a late response (10 h after the challenge) and dermographism after the SPT. Of the 3 patients only one had a positive RAST to pectin [119].

**Table 1:** Immunomodulatory effects of pectin and impact on the manifestation of immune responses

Type of pectin	Model	Microbiota	SCFAs	Effects	Ref
Apple	In vivo: Murine HDM asthma model (Fed with 30% pectin)	↑ Bacterioidetes ↑ Bifidobacteriaceae ↓ Firmicutes	↑ Acetate ↑ Propionate ↑ Butyrate	Reduced allergic airway response Changes in intestinal and lung microbiota Increment in SCFAs was proportional to the high-fiber diet	[98••]
Apple	In vitro: Pepsin digestion of fruit derived allergens			The presence of pectin in the fruit matrix influences the digestibility of food allergens High pectin content was associated with protection against digestion of kiwi allergens	[115]
Apple	In vivo: Obesity rat model	↑ Bacterioidetes ↑ Firmicutes		Pectin consumption reduced weight gain, inflammation, and metabolic endotoxemia	[132]
Apple	In vivo: Obesity rat model		↑ ↑ ↑ Acetate ↑ ↑ ↑ Propionate	Pectin consumption reduced food intake and reduced body fat loss by 23% Increased SCFAs in cecum, but decreased branched-chain fatty acids	[134]
Apple and citrus	In vivo: Murine model of oral tolerance			CP oral administration inhibited immune hypo-responsiveness induced by OVA feeding CP-fed mice showed increased levels of IgG1 and IgE in sera and increasing OVA immunogenicity CP treatment increased the levels of TNF-α and IFN-γ in peritoneal macrophages AP showed no effect on the studied factors	[120]
Asian pear	In vivo: Murine model of asthma			Administration of Asian pear pectin suppressed asthmatic reactions in sensitized mice as observed in the tracheal smooth muscle IFN-γ increased in the BAL of pectin-treated mice while IL-5 diminished Treatment with pectin prior to sensitization reduced the OVA sIgE by 70%	[122]
Citrus (different DE)	In vitro/in vivo: Human cell lines/doxorubicin-induced Ileitis in mice		SCFAs not enhanced in the murine model	DE-dependent inhibition of TLRs TLR2 inhibited by direct binding to TLR2 ectodomain ↓ DE—> ↑ effect	[105]
Citrus	In vivo: Mice fed with high-fat diet + pectin	↑ Bacteroides ↑ Parabacteroides ↑ Olsenella ↑ Bifidobacterium	↑ Acetate ↑ Propionate	Pectin consumption influenced the gut microbiota and SCFA production Pectin showed hepatoprotective activity, regulating lipid metabolism, inflammation, and antioxidant production	[176]
Citrus (LMP and HMP)	In vivo: Germ-free rats fed with pectin	↑ Bacteroides	↑ Acetate ↑ Propionate ↑ Butyrate	LMP fermented faster than HMP both in vitro and in vivo Pectin-fed rats produced higher levels of SCFAs and had higher ileum and colon weight	[138]
Citrus	Case report: SPT using commercial pectin			Anaphylaxis after consumption of a pectin-containing smoothie Positive RAST (more than 100 KU/L) to cashews and pistachios Possible cross-reactivity between pectin and cashews and pistachio proposed	[110]
Citrus	Case report: Occupational rhinitis, conjunctivitis, and contact urticaria to pectin			SPT positive to cashew, pistachio, and pectin Positive blood basophil histamine release from cashew, pistachio, and pectin Negative RAST to pectin, but positive to orange, cashew, and pistachio Positive immunospot to cashew, pistachio, and pectin No oral challenge performed Cross-reactivity between pectin and cashews proposed	[114]
Citrus	Case report: Occupational asthma			Subject reported shortness of breath while working with pectin After inhalation challenge with pectin developed chest tightness, shortness of breath and wheezing SPT positive to pectin Reported allergy to cashews Normal total IgE, sIgE was not detected Increased pectin sIgG4 reported	[117]
Source not indicated	Case report: Occupational asthma			Reported wheezing, cough, rhinorrhea after pectin exposure SPT positive to pectin as well as to other common allergens as HDM, grass, ragweed Reduced peak flow at work (where pectin was used) than at home Assumption as pectin as elicitor agent	[118]
Source not indicated	Case report: Occupational asthma (3 reports)			3 patients reported shortness of breath, cough, and nasal symptoms 2 patients showed immediate responses in lung function after pectin inhalation challenge, and positive pectin SPT 3rd patient showed late response after pectin inhalation challenge 2 patients had negative sIgE- and sIgG4-pectin antibodies. Only 1 patient was RAST positive to pectin Authors suggests that type I hypersensitivity has a role in the development of pectin-induced asthma	[119]
Citrus, rhamnogalacturonan-I (RG-I)-enriched pectin (WRP) and the depolymerized fraction (DWRP)	In vivo: Mice e.g., supplemented with pectin	DWRP: ↑ Bifidobacterium ↑Lactobacillus WRP: ↑Ruminococcaceae ↓Actinobacteria	CP and WRP: ↑ Acetate	All pectins increased SCFA concentrations in cecum WRP and DWRP beneficially modulated gut microbiota in a structure-dependent way	[155]
Citrus, sugar beet and soy	In vivo: Rats fed with 3% pectin	↑ Lactobacillus ↑ Lachnospiraceae	CP (LMP) and soy pectin: ↑ Propionate ↑ Butyrate	LMP is more easily fermented than HMP and tends to result in the production of higher amounts of SCFAs	[156]
Citrus and MCP	In vivo: Pectin in drinking water (murine)			Splenocytes of citrus pectin and MCP-treated mice showed increased levels of IL-17, IFN-γ, and TNF-α	[108]
MCP with different DE	In vitro: Murine macrophages			Pectin inhibited iNOS and COX-2 mRNA expression in LPS-activated macrophages ↑ DE—> ↑ inhibition Pectin with 90% DE: Inhibited MAPK phosphorylation, IKK kinase activity, and NF-κB and inhibited LPS binding to cells	[106]
MCP	In vitro: Human blood samples			Activated CD8^+^ T cells, B cells, and NK-cells in dose-dependent manner	[107]
Long-chain fructo-oligosaccharides (FOS)	Randomized controlled clinical trial	↑ Bifidobacteria ↓ Bacteroides ↓ Clostridia		Infant formulae supplemented with FOS increased the bifidobacteria proportions and reduced the alteration of fecal microbiota after diet cessation	[139]
Strawberry	Case report Anaphylaxis after strawberry-flavored yogurt			Anaphylaxis 30 min after ingestion of strawberry-flavored yogurt Pectin considered most likely trigger of allergic reaction Positive SPT to pectin and almond, cashew, hazelnut, pecan, pistachio, and walnut Possible cross-reactivity to cashew and pistachio suggested	[111]
Strawberry	In vitro Human PBMCs and U266 cell line			Alkali soluble pectin showed IgE-suppressive activity in human PBMCs and U266 (human myeloma) cells	[112]

*AP* apple pectin, *CP* citrus pectin, *HDM* house dust mite, *MCP* modified citrus pectin, *SCFA* short-chain fatty acids, *OVA* ovalbumin

In vivo, oral administration of citrus pectin prevented the induction of immune tolerance induced by feeding of a high dose of OVA [120]. Citrus pectin feeding inhibited the development of the oral tolerance in the OVA-treated mice. Mice fed with pectin showed similar titers of antigen-specific serum IgG and similar levels of delayed-type hypersensitivity responses as those animals not tolerant. Here, citrus pectin increased the levels of serum OVA-specific IgG1 and IgE [120].

In contrast, other studies reported a beneficial effect of pectin on allergic sensitization by alteration of the intestinal microbiota [39]. Increased numbers of beneficial bacteria like *Bifidobacterium* and the higher production rate of bacteria-derived SCFAs were suggested to lower the risk of food allergies [121]. Pectin containing more than 80% galacturonic acid residues was found both to decrease macrophage activity and inhibit delayed-type hypersensitivity reactions [50]. Moreover, alkali-soluble pectin suppressed IgE production in a human myeloma cell line in vitro [112]. Other results also indicate that administration of Asian pear pectin-sol (a pH and enzymatically modified pectin in colloidal dispersion) in sensitized mice suppressed allergic asthmatic reactions [122]. However, little is known whether pectin from different plant sources and different degrees of esterification exert distinct immune modulating properties.

### Modulation of Gastrointestinal Microbiota by Pectin

Pectin and POS are fermented in the colon by different bacterial genera such as *Bifidobacteria*, *Lactobacilli*, *Enterococcus*, *Eubacterium rectale*, *Faecalibacterium prausnitzii*, *Clostridium*, *Anaerostipes*, and *Roseburia* spp*.* in order to promote their growth [68, 69, 123, 124]. The degradation of pectin is facilitated by different bacteria-derived enzymes such as pectinases, methylesterases, acetylsterases, and lyases [125–127], generating different POS that will vary depending on microbiota composition and pectin structure [69, 100•, 128–132].

As mentioned before, the biological properties of both pectin and POS depend on different features such as molecular weight (MW) and type and structure of constituents (e.g., galacturonic acid or rhamnose) and DE [51, 128]. It is reported that pectin from orange, lemon, and sugar beet with high degree of methylation increase the colonization by *Bifidobacterium*, *Bacteroides*, and *Eubacterium* species, while the POS derived from the same sources promoted the presence of *Bifidobacterium species, Bacteroides*, and *Lactobacillus* when compared to the primary pectin [133••]. Pectins with high DE are degraded slower than the ones with lower DE and are reported to remain in the intestine for up to 24 h [134–136], while pectins with lower DE were easily metabolized [135-138]. In vitro fermentation studies of pectin and POS using human fecal samples have shown an increase in the number of *Bifidobacteria*, *Eubacterium rectale*, *Clostridium coccoides,* and *Bacteroides prevotella*, with an elevated production of acetate, propionate and butyrate (summarized in Table 2) [40, 129]. The shift observed in the microbiota correlates with clinical effects of POS: POS-supplemented infant formulae was shown to both increase the number of *Bifidobacteria* and *Lactobacilli* and minimize the alteration of fecal microbiota after cessation of breast-feeding [139].

**Table 2:** Pectin effects reported using in vitro fermentation systems

Type of pectin	Bacterial source	Microbiota	SCFAs	Summary	Ref
Apple	Human feces	↑ Bacterioidetes ↑ Firmicutes Strong and highly specific enrichment of *Eubacterium eligens*	Pectin avoided changes in SCFAs (acetate, propionate, and butyrate) production by pH changes	pH did not influence pectin fermentation by fecal microbiota Pectin fermentation generated a greater microbiota diversity, as probably the chemical complexity generated multiple nutritional niches	[68]
Apple and citrus	Human feces		↑ Acetate ↑ Propionate ↑ Butyrate	The degree of methylation is the most important factor influencing the fermentation in the colon HMP are faster and more extensively fermented than LMP	[144]
Apple and sugar beet	Human feces	↑ Bifidobacteria	↑↑ Butyrate ↑ Acetate	↑ SCFA production after pectin fermentation The type of carbohydrate fermented is related to dynamic changes in the gut microbiota composition, affecting SCFAs production	[131]
Apple POS	Human feces	↑ Bifidobacteria ↑ Lactobacilli ↓ Bacteroides ↓ Clostridia	↑ Acetate ↑ Propionate	POS showed increased prebiotic effects when compared to the original pectin	[40]
Citrus	Rumen bacteria		↑↑↑ Acetate ↓ Butyrate	High fermentation of acetate and low levels of butyrate, lactate, succinate, and fumarate by *B. fibrisolvens* and *P. ruminicola*	[123]
Citrus	Human feces		↓ Butyrate	Pectin fermentation by butyrate-producing cells generated the lowest production of butyrate (56%) out of 5 carbohydrate sources	[124]
Citrus	Human feces	↑ Bifidobacteria ↑ *Eubacterium rectale*	↑ Butyrate	Citrus pectin and POS modulated bacteria composition, increasing the ratio of beneficial bacteria and butyrate production	[129]
Citrus	Human feces	↑ Lachnospira ↑ Dorea ↑ Clostridium ↑ Sutterella	↑ Acetate ↑ Butyrate	Pectin fermentation increases bacterial species of the Clostridium cluster XIV and stimulates the production of acetate and butyrate	[178]
Citrus and sugar beet (9 types)	Human feces	↑ Bacterioidetes ↑ Enterobacteriaceae	↑ Propionate by HMP or by high content of Rha	Gut microbiota can be modulated by pectin, depending on the structural features DE probably the most important parameter (correlation of bacterial taxa with DE) Propionate is mainly a product of HMP fermentation	[100•]
Citrus, sugar beet and POS	Human feces	↑ Bifidobacteria ↑ Lactobacilli ↑ Faecalibacterium ↑ Roseburia	↑ Acetate ↑ Propionate ↑ Butyrate	POS showed better prebiotic effects than pectin itself SCFA production was similar between different POS, but higher than SCFA production by pectin The ratio between SCFAs varies depending on the POS type	[136]
Orange POS	Human feces	↑ Bifidobacteria ↑ Lactobacilli	Concentration profile: acetate > butyrate > propionate	Pectin induced comparable SCFAs generation as FOS, but with a reduced butyrate generation	[69]
Sugar beet POS	Human and pig feces	↑ Bacterioidetes	↑↑↑ Acetate ↑↑ Propionate ↑ Butyrate ↓ Valerate	Sugar beet POS were completely fermented by human and pig fecal microbiota Paralleled by an increase in SCFA production	[130]

*POS* pectin oligosaccharides, *SCFA* short-chain fatty acids, *DE* degree of esterification

It is well accepted that an increase in bacteria-derived SCFAs promotes a protective effect in the intestine [74, 140–142]. In line with this, the composition of the gut microbiota can influence the development of the immune system and homeostatic processes at which the dysbiosis of microbial composition can increase the susceptibility for immune-mediated diseases, like asthma or allergy (reviewed in [143•]).

**Fig. 4:**
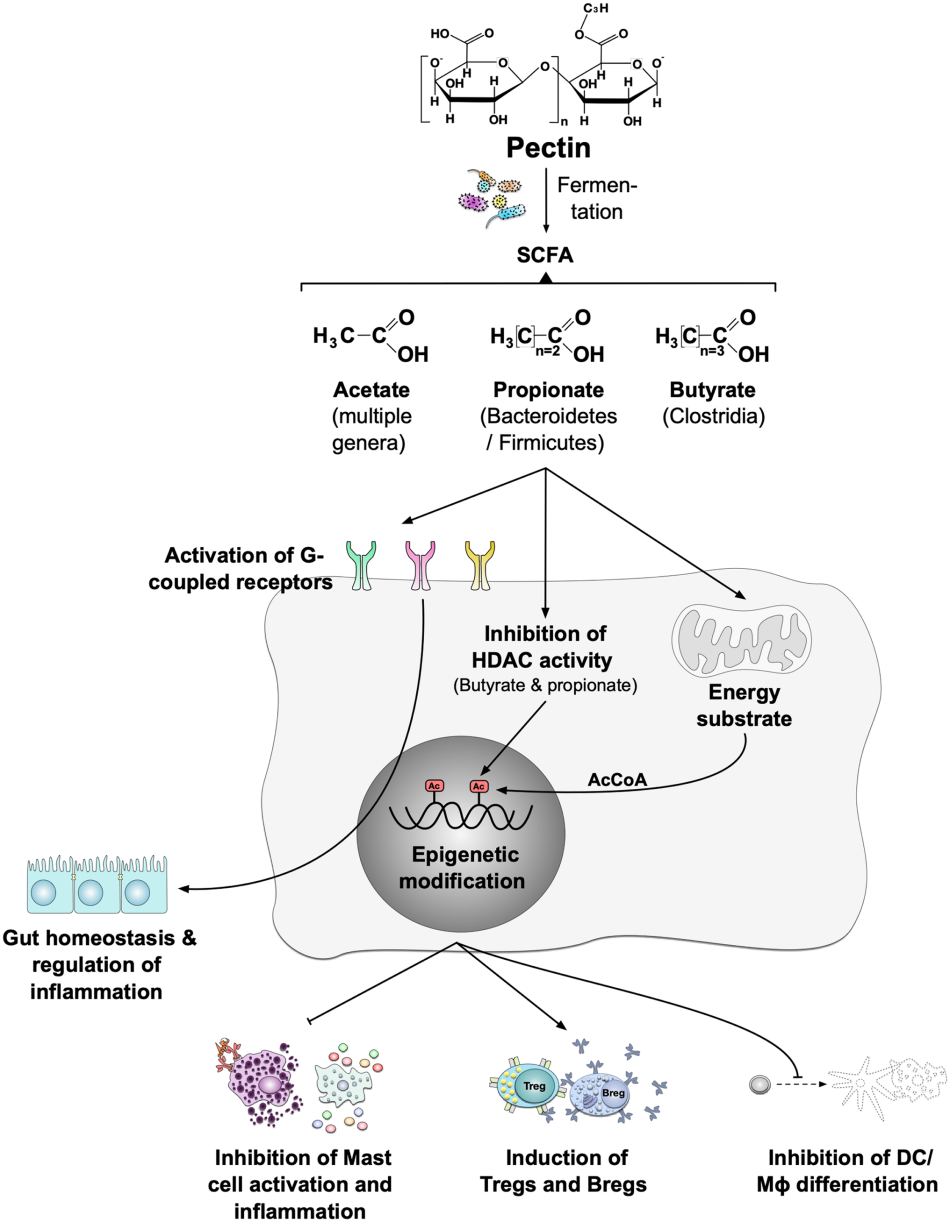
Immune modulation by pectin-derived SCFAs. Pectin fermentation by gut microbiota leads to the production of SCFAs. Different genera can generate different SCFAs. For example, acetate can be produced by many different genera; propionate is mainly produced by *Bacteroidetes* and *Firmicutes*, while butyrate is mainly produced by *Clostridia* species. SCFA bind “metabolite-sensing” G-protein-coupled receptors such as GPR41, GPR43, GPR109A, and olfactory receptor (Olfr)-78. These receptors promote the gut homeostasis and the regulation of inflammatory responses. GPRs and their metabolites influence T_reg_ activation, epithelial integrity, gut homeostasis, DC biology, and IgA antibody responses. Through their inhibition of HDAC expression or function, SCFAs also influence gene transcription in many cells and tissues

Compared to LMP, the fermentation of HMP increased the abundance of *Prevotella* and *Ruminococcus* species and was paralleled by higher levels of the SCFA propionate [100•, 144]. Particularly, levels of *Ruminococcus*-derived propionate are enhanced when stimulated with rhamnose and fucose, both of which are structural subunits of pectin. This indicates that a higher production of propionate could relate with a high pectin content of rhamnose and fucose, as well as its slower fermentation process [145–147]. The SCFA acetate is reported to be produced by many different genera, but mainly by *Bifidobacteria* and *Lactobacilli* (Table 2) [148, 149]. Propionate is mainly produced by *Bacteroidetes* and *Firmicutes* (via the succinate pathway) [146, 150], while butyrate is produced by *Eubacterium rectale*, *Roseburia intestinalis*, *Faecalibacterium prausnitzii*, and some *Clostridia specie*s (both from pyruvate via butyryl CoA:acetate CoA transferase and directly from acetate (Fig. 4) [151, 152].

### Pectin-Induced Short-Chain Fatty Acids

Dietary fibers, including pectin, are fermented by commensal gut bacteria, which produce metabolites including SCFAs, particularly butyrate, propionate, acetate, and pentanoate (valerate) [36, 74, 100•, 138, 144, 153, 154]. Fermentation of every structurally distinct pectin induces specific profiles of SCFAs in the gastrointestinal tract [100•, 103, 138, 144, 155, 156]. In addition, the generation of SCFAs is strongly dependent on substrate availability, microbiota composition, and intestinal transit time [157].

SCFAs can mediate anti-inflammatory effects by (I) enhancing the frequency of immune regulatory T_reg_ cells in the intestine [158], (II) inhibiting Th2-mediated airway diseases in mice [159], (III) stimulating epithelial cell growth, (IV) suppressing APC activation, and (V) maintaining a low pH in the intestine (which inhibits pathogen growth) [103, 157]. Vice versa, a dysfunctional microbiome with a reduced capacity to produce SCFAs is prone to the development of allergic diseases [160]. SCFAs likely modulate immune responses by three different mechanisms: (I) directly activating G-coupled receptors, (II) inducing epigenetic modifications by inhibiting histone deacethylases (HDAC), and (III) serving as energy substrates for both immune and non-immune cells (Fig. 4) [121, 161•].

The molecular mechanisms by which SCFAs are involved in the “diet-gut microbiota-physiology axis” have been explored recently. SCFAs bind “metabolite-sensing” G-protein-coupled receptors such as GPR41 (affinities: acetate = propionate > butyrate), GPR43 (butyrate = propionate > acetate), GPR109A (butyrate), and olfactory receptor (Olfr)-78 (propionate = acetate) (reviewed in [10]). These receptors play fundamental roles in the promotion of gut homeostasis and the regulation of inflammatory responses (Fig. 4). For instance, GPRs and their metabolites influence T_reg_ activation, epithelial integrity, gut homeostasis, DC biology, and IgA antibody responses [6, 162]. Through the inhibition of HDAC expression or function, SCFAs also influence gene transcription in many cells and tissues [163]. GPR41 and GPR43 are expressed on epithelial cells, macrophages, and DCs [6]. Here, GPR43 is engaged in suppression of bacterial invasion into the tissue, prevention of inflammation, intestinal carcinogenesis (reviewed in [164]), and allergy [165]. GPR109A is expressed primarily in adipocytes and immune cells as DCs, neutrophils, macrophages, intestinal and colonic epithelial cells [166–169]. The GPR109A/butyrate axis is reported to suppress the tumor development and progression and the LPS-induced NF-ĸB activation [170], as well as an anti-inflammatory role by modulation factors like CCL5, MCP-1, and TNF-α [171–174].

High levels of both butyrate and propionate in early life are associated with protection against atopy [175••]. Trompette et al. reported that feeding mice with either a high pectin diet or supplementation of propionate enhance levels of SCFAs and protected against allergic lung inflammation [98••]. These protective effects were shown to be independent of either T_reg_ induction, activation status, or recruitment of dendritic cells to draining lymph nodes [98••]. In their model, propionate treatment enhanced hematopoiesis of common DC precursors and macrophage-DC precursors [98••].

In line with these anti-inflammatory properties of pectin, oral ingestion of pectin for 8 weeks in a mouse model of non-alcoholic fatty liver disease MAPK dependently improved lipid metabolism and decreased both oxidative stress and inflammation [176]. In this study, pectin feeding dose-dependently increased levels of both acetic and propionic acids and relative abundance of *Bacteroides*, *Parabacteroides*, *Olsenella*, and *Bifidobacterium* species in the gut of pectin-fed mice [176].

In rats, Fukunaga et al. reported pectin supplementation (2.5% pectin for 15 days) to result in significant increases in the length, weight, and number of Ki-67-positive cells in the ileum, cecum, and colon [177]. While in this model, pectin supplementation did not affect the composition of the cecal microbial flora, cecal SCFA content and plasma glucagon-like peptide-2 in small intestine were significantly increased [177].

Larsen et al. characterized the relationship between structural properties of pectin and their ability to modulate both composition and activity of gut microbiome [100•]. In vitro fermentation of nine structurally diverse pectins from citrus fruits, sugar beet, and a pectin derivative rhamnogalacturonan I using a TIM-2 colon model under anaerobic conditions was performed [100•]. Here, cumulative production of total SCFAs as well as propionate was highest for fermentation of high methoxyl pectin (including rhamnogalacturonan I), while acetate levels were similar for all investigated pectins [100•]. This increased production of SCFAs by RG-I was independently confirmed in rats fed with RG-I-enriched diets [155]. Interestingly, bacterial populations associated with human health (e.g., *F. prausnitzii*, *Coprococcus*, *Ruminococcus*, *Dorea*, *Blautia*, *Oscillospira*, *Sutterella, Bifidobacterium*, *Christensenellaceae*, *P. copri*, and *Bacteroides* spp.) were either increased or decreased depending on the used pectin, suggesting that microbial communities in the gut can be specifically modulated using different pectins [100•].

Finally, Bang and co-authors performed in vitro fermentation (1% pectin from citrus peel) experiments using the feces of three Korean donors in order to investigate pectin-induced changes in the gut microbiome and SCFA production [178]. Pectin fermentation commonly increased the frequencies of pectin-degrading bacterial species belonging to the Clostridium cluster XIV (*Lachnospira*, *Dorea*, and *Clostridium*) and *Lachnospira* paralleled by an increase in acetate (starting as early as 6 h after start of incubation), as well as propionate and butyrate (after 12 h of incubation) [178].

## Summary

The role of dietary fiber pectin in the development of allergic reactions is controversial. Several clinical reports indicate the manifestation of allergic reactions after pectin consumption, probably attributed to cross-reactivity between pectin and allergens. Moreover, pectin was also described to prevent the digestion of allergens in the stomach, facilitating intact allergen molecules to reach the gut and to induce allergic reactions.

However, others showed direct and indirect immune modulating effects of pectin. A broad set of evidence describing application of pectin to induce a shift to beneficial microbiota and an increase in the levels of SCFAs, both of which have been associated with reduced inflammatory and allergic reactions in vitro and in vivo has been provided. As bacterial populations associated with human health were either increased or decreased by different pectins, it is likely that bacterial communities in the gut can be specifically modulated by pectin application. Pectin is able to directly interact with immune cells such as DCs and macrophages by electrostatic interactions with TLR2, thereby inhibiting the pro-inflammatory TLR2-TLR1 pathway, while not affecting the TLR2-TLR6 tolerogenic pathway. Also, it is able to bind LPS affecting its binding to TLR4. Other cell types such as T cells, B cells, and NK cells are also activated by pectin, while iNOS and COX-2 expression are inhibited in peritoneal macrophages by inhibition of the IKK activity, MAPK phosphorylation, and NF-κB activation, suggesting an anti-inflammatory property.

Considering the data reviewed here, it is tempting to speculate that dietary fiber including certain pectin can be considered for prophylactic intervention targeting microbiota in immune-related diseases, such as allergies. The immune modulating effect seems to be dependent on the structure and source of the pectin. In particular, the direct immune modulating mechanism by pectin and pectin-derived products generated by food processing and fermentation remain to be investigated.

## Abbreviations

Ara: Arabinose
DE: Degree of esterification
FA: Food allergy
FOS: Fructooligosaccharide
Gal: Galactose
GalA: Galacturonic acid
GFA: Fructo- and acidic-oligosaccharide
GOS: Galactooligosaccharide
GPR: G-protein-coupled receptor
HDAC: Histone deacetylase
HMP: High methoxy pectin
LMP: Low methoxy pectin
MCP: Modified citrus pectin
OVA: Ovalbumin
pAOS: Pectin-derived acidic oligosaccharides
POS: Pectic polysaccharides
Rha: Rhamnose
SCFAs: Short-chain fatty acids
